# Examining the value of body gestures in social reward contexts

**DOI:** 10.1016/j.neuroimage.2020.117276

**Published:** 2020-11-15

**Authors:** Elin H. Williams, Laura Bilbao-Broch, Paul E. Downing, Emily S. Cross

**Affiliations:** a School of Psychology and Clinical Language Sciences, University of Reading, Reading, England; b Korea Institute for Science and Technology, University of Science and Technology, Seoul, South Korea; c Wales Institute for Cognitive Neuroscience, Bangor University, Bangor, Wales; d Institute of Neuroscience and Psychology, University of Glasgow, Glasgow, Scotland; e Department of Cognitive Science, Macquarie University, Sydney, Australia

**Keywords:** Biological motion, Body gestures, Reward value, Social motivation, Reward anticipation

## Abstract

Brain regions associated with the processing of tangible rewards (such as money, food, or sex) are also involved in anticipating social rewards and avoiding social punishment. To date, studies investigating the neural underpinnings of social reward have presented feedback via static or dynamic displays of faces to participants. However, research demonstrates that participants find another type of social stimulus, namely, biological motion, rewarding as well, and exert effort to engage with this type of stimulus. Here we examine whether feedback presented via body gestures in the absence of facial cues also acts as a rewarding stimulus and recruits reward-related brain regions. To achieve this, we investigated the neural underpinnings of anticipating social reward and avoiding social disapproval presented via gestures alone, using a social incentive delay task. As predicted, the anticipation of social reward and avoidance of social disapproval engaged reward-related brain regions, including the nucleus accumbens, in a manner similar to previous studies’ reports of feedback presented via faces and money. This study provides the first evidence that human body motion alone engages brain regions associated with reward processing in a similar manner to other social (i.e. faces) and non-social (i.e. money) rewards. The findings advance our understanding of social motivation in human perception and behavior.

## 1 Introduction

Humans are an inherently social species, and from an early age, we spend much of our daily lives engaging, interacting, and communicating with others. This strong motivation to engage socially directs our attention to social signals, guides us to participate in behaviors that help us to establish, maintain, and enhance our relationships with others, and allows us to enjoy social interactions and to find them rewarding (Chevallier et al., 2012). Social stimuli, such as dynamic and static displays of human faces and bodies, are valued by participants (Dubey et al., 2015; Williams and Cross, 2018; Hayden et al., 2007) and engage attention more than non-social stimuli (Williams et al., 2019; Chakrabarti et al., 2017; Gray et al., 2018). For example, typically developed participants assign a higher value to smiling faces with direct gaze (Dubey et al., 2015) and human bodies moving naturally (Williams and Cross, 2018; Williams et al., 2019), compared to less social stimuli. Social stimuli are suggested to be rewarding as they provide an abundance of valuable information to the perceiver, such as an interaction partner's identity, age, gender, and even their emotions and intentions (Hahn et al., 2016). Such information allows a perceiver to decide whether to engage in, or to avoid, social interaction with another individual.

Individuals also make daily decisions to direct behavior towards positive stimuli such as social rewards (Fellows, 2004). Social reward processing has long been investigated behaviorally (Dubey et al., 2015; Williams and Cross, 2018; Williams et al., 2019; Chakrabarti et al., 2017; Hahn et al., 2016), and recent studies have also investigated the underlying neural mechanisms of social rewards in order to gain a greater understanding of reward processing in the typical population, as well as in individuals with social difficulties that are the hallmark of an autism spectrum condition (ASC) diagnosis (Kohls et al., 2013, 2011; Spreckelmeyer et al., 2009; Rademacher et al., 2010). These studies report that positive social feedback provided via faces is associated with activation of the same reward-related brain regions, such as the ventral (nucleus accumbens; NAcc) and dorsal striatum (caudate nucleus and putamen), the amygdala, and the orbitofrontal cortex (OFC), as non-social incentives, such as money (Spreckelmeyer et al., 2009). Similar brain regions are also activated in anticipation of food (O'Doherty, Deichmann, Critchley, & Dolan, 2002), drugs (Childress et al., 1999), and sex (Childress et al., 2008).

A prominent experimental paradigm developed by Knutson et al. (2000) known as the Monetary Incentive Delay (MID) task facilitates the assessment of the neural mechanisms underlying reward processing, while also allowing researchers to dissociate reward anticipation (‘wanting’) from reward consumption (‘liking’), using functional magnetic resonance imaging (fMRI). The MID task has recently been modified to assess responses to social incentives via the Social Incentive Delay task (SID; Kohls et al., 2011, 2013; Rademacher et al., 2010; Spreckelmeyer et al., 2009). In these tasks, participants are presented with a brief cue, followed by a variable interval; they then must respond to a white target square presented briefly. Participants receive feedback (monetary, social, or otherwise) regarding whether they responded rapidly enough. The duration of the presentation of the target is adjusted to keep performance at a desired below-ceiling level.

Research comparing neural responses for the cued anticipation of social rewards (i.e. faces) compared to monetary rewards in typically developing participants, using incentive delay tasks like these, reports that anticipation of both social and monetary rewards activates the dopaminergic mesocorticolimbic reward circuitry, including the ventral striatum (Spreckelmeyer et al., 2009; Dichter et al., 2012; Gasic et al., 2009). In both MID and SID tasks, NAcc activity increases parametrically with expected reward value and subjective preference (Knutson et al., 2000; Spreckelmeyer et al., 2009). Therefore, although NAcc activation serves as a general mediator of reward prediction regardless of modality, response amplitude is mediated by the saliency of the reward.

Recently, studies have begun to investigate in more depth the neural mechanisms underpinning the companion behavior to reward seeking: namely, the motivation to avoid punishment. Studies have investigated avoidance of punishment in the form of monetary loss (Carter et al., 2009; Delgado et al., 2009) and social disapproval (Kohls et al., 2013). As with monetary gains and social approval, avoidance of monetary loss and social disapproval is also associated with activation of the NAcc. The NAcc is, therefore, suggested to be involved in goal directed behavior, motivating the participant to obtain rewards and avoid punishment, whether social or non-social in nature.

While we are beginning to gain a fuller appreciation of the value of social stimuli to human behavior, one shortcoming of previous studies that have investigated the neural mechanisms underlying the anticipation of social reward is their reliance on static images of faces to serve as social rewards (Risko et al., 2012). In the real world, most of our social interactions involve perceiving and interacting with other people moving around us in a dynamic and constantly shifting social landscape. In a recent study, Kohls et al. (2013) took a step towards establishing the importance of dynamic social cues by developing a set of dynamic stimuli featuring non-verbal feedback provided via faces and body gestures presented together. These face and body gestures were then used to convey social approval (reward) or social disapproval (punishment) in the SID. Using these dynamic stimuli, Kohls and colleagues reported engagement of the NAcc, comparably to previous studies using static images, when participants anticipated social approval or were avoiding social disapproval. These findings extend prior findings that have used non-social incentives (such as monetary gain and loss) in MID tasks, by demonstrating the bivalent activation pattern of the NAcc in response to anticipated social reward and avoidance of social disapproval.

While the study by Kohls et al. (2013) took an important first step towards establishing the reward value of dynamic social cues as these researchers presented videos of faces and body gestures together, it remains unclear what the value of social feedback provided via body gestures alone is. This is an important question because body gestures and expressions are particularly informative social cues that perceivers are able to extract meaning from. We can read a wealth of social cues from other people's bodies even when we lack visual access to the face (such as seeing someone from a distance or from behind; de Gelder, 2006; Greven et al., 2019), and previous research suggests that the body reveals emotion more accurately than the face (Aviezer et al., 2012). Face perception work has led to a generally agreed neurocognitive model, whereas that prospect remains distant for body perception. Learning more about body perception in isolation from faces helps us to draw parallels, or make distinctions, between these two classes of social stimuli.

Previous work demonstrates that participants value videos of human figures moving biologically more than videos featuring less social, robot-like motion (Williams and Cross, 2018), and natural human motion also engages attention more than other less social motion and induces autonomic arousal (Williams et al., 2019). A rich literature documents how body movements provide valuable non-verbal information to perceivers (de Gelder, 2006; Meeren et al., 2005; Van den Stock, Righart, and de Gelder, 2007; Rosenthal et al., 1979; Johnson and Shiffrar, 2012; Argyle, 2013; Yovel and O'Toole, 2016). With the present study, therefore, we aimed to assess whether the anticipation of obtaining social rewards via body gestures alone leads to similar engagement of brain structures associated with reward anticipation (i.e. the ventral and dorsal striatum, amygdala and OFC) as has been reported for faces. Additionally, due to evidence of NAcc engagement during anticipated avoidance of social disapproval (Kohls et al., 2013), we included a social disapproval condition to confirm that brain regions associated with social reward processing are also engaged when avoiding social disapproval, as conveyed by body gestures.

Using a modified version of the SID, participants completed a task that included social approval and social disapproval (Kohls et al., 2013), wherein positive or negative feedback was provided either via body gestures or via text (in a control condition). We hypothesized that both the anticipation of approval and avoidance of disapproval for both body motion and text trials would result in greater reward region activation compared to neutral feedback. Additionally, we hypothesized that social cues to approval or avoidance of disapproval provided via body motion should be more salient than cues provided via text, and thus we expected greater engagement of brain regions associated with reward processing, particularly in the NAcc, during anticipation of approval and avoidance of disapproval presented via body gestures compared to text. This study focused on the reward anticipation (‘wanting’) rather than the reward consumption (‘liking’) aspect of social reward following Kohls et al. (2013) .^1^

## 2 Materials and method

### 2.1 Open science statement

Consistent with recent proposals (Simmons et al., 2011, 2012), we report all data exclusions, all manipulations, and all measures in the study. In addition, following open science initiatives (Munafò et al., 2017), the data and examples of stimuli associated with this study are freely available online (https://osf.io/qph9m/?view_only=fb20aef4985a405081ef62cb610d860e). By making the data available, we enable others to pursue tests of alternative hypotheses, as well as more exploratory analyses.

### 2.2 Participants

Thirty-two healthy young-adult volunteers were recruited from Bangor University's student participant panel and from the local community. Participants received course credits or £20 for their time. The sample size was selected based on a similar experiment conducted by Kohls et al. (2013). One participant was excluded from the final sample due to excessive head motion during scanning (more than 3 mm of translational motion in the *x, y*, and *z* planes during multiple runs), and two participants were excluded due to having average reaction times of more than 2 standard deviations from the mean, leaving a final sample of 29 participants (17 females, *M*_age_ = 23.38, *SD* = 2.53). However, two runs were discarded from one participant, and one run was discarded from another participant due to excessive head motion, and some volumes were discarded from three participants due to excessive motion at the beginning, or towards the end, of a run. All participants had normal or corrected-to-normal vision, were right-handed, and reported no history of neurological illness. Bangor University and the Bangor Imaging Unit provided ethical approval (Ethical Approval Code: 2017-15913), and all participants provided written informed consent prior to participating.

### 2.3 Stimuli

#### 2.3.1 Video stimuli

**Fig. 1:**
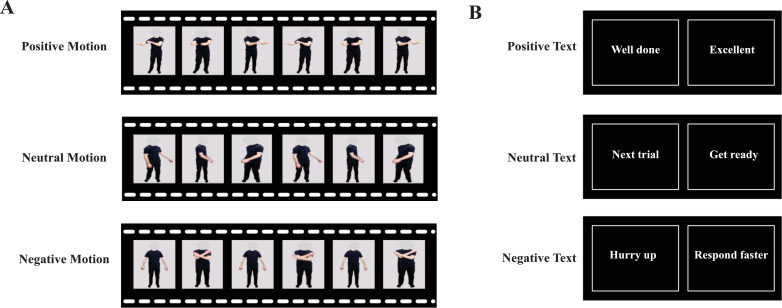
(A) Video stills from the positive, neutral, and negative stimulus categories. (B) Text examples from the three stimulus categories.

Three categories of video stimuli were developed for the experiment. Two actors (1 female and 1 male) were instructed to perform a series of positive (e.g. thumbs up), negative (e.g. thumbs down), and neutral body movements (e.g. clicking fingers) in front of a green screen (see Supplementary Videos for examples). For the final video stimuli, the green screen was replaced with a grey background, and a mask (consisting of a grey oval) was placed over the actors’ faces to ensure that the only channel for emotional expression was the actors’ body motion (see Fig. 1A).

These stimuli were chosen following an online validation study conducted via Qualtrics (Provo, Utah) with an independent group of 32 participants (19 females, *M*_age_ = 19.63, *SD* = 1.50) prior to the start of the fMRI study. In this online validation study, participants were asked to rate the emotional valence of 104 videos on a sliding scale from 0 to 100 (with anchors 0 = negative, 50 = neutral, 100 = positive). From this original stimulus set of 104 videos, we selected the 10 video stimuli (5 female, 5 male) that were rated the most positively (*M* = 77.09, *SD* = 8.49), the 10 video stimuli that were rated the most negatively (*M* = 22.46*, SD* = 8.45), and the 4 (2 male, 2 female) video stimuli that received the most neutral ratings (*M* = 47.61, *SD* = 3.50) to compose the final video stimulus set to be used during scanning (Fig. 1A & Fig. 5). A repeated-measures ANOVA confirmed that the three video categories were rated as significantly differently from each other (*F*(1.262, 39.124) = 360.29, *p* < 0.001) (all Sidak-corrected comparisons had *p*-values of <0.001).

The fMRI study featured 24 video stimuli in total, composed of the male and female actor each performing the same 12 distinct movements. To ensure that any differences in brain activity revealed during observation or anticipation of positive and negative movements were not due to low-level features, such as the number or scale of movements featured within a video category, we calculated a measure of overall pixel displacement from frame to frame for each video and then compared this across video categories. This so-called “motion energy” was quantified for each video using MatLab (R2015b; for a more complete description of the motion quantification algorithm, please see Cross et al., 2012). This analysis revealed no reliable differences in motion energy between the three video categories (*F*(2, 21) = 0.639, *p* = 0.538).

#### 2.3.2 Text stimuli

Text stimuli were piloted in the same way as the video stimuli; participants completed an online rating pilot experiment via Qualtrics, and rated the emotional content of 83 text stimuli using a sliding scale from 0 to 100, with 0 being the most negative, and 100 being the most positive. The 10 most positively rated text stimuli (*M* = 81.45, *SD* = 9.35), the 10 most negatively rated text stimuli (*M* = 26.98, *S*D = 11.35), and the two stimuli that were rated the most neutrally (*M* = 49.56, *SD* = 10.52) were chosen (Fig. 1B & Fig. 5). This resulted in a total of 22 distinct text stimuli for the final experiment. A repeated-measures ANOVA confirmed that the three text categories were rated as significantly differently from each other (*F*(1.687, 52.287) = 215.270, *p* < 0.001) (all Sidak-corrected comparisons had *p*-values of < 0.001).

Paired t-tests between stimulus category (positive, negative, and neutral) and feedback modality (motion and text) confirmed that the three categories were not significantly different from each other in the motion and text stimulus sets (all *p*-values > 0.05).

### 2.4 Procedure

#### 2.4.1 Screening session

Participants were invited to the laboratory prior to the fMRI testing session to ensure fMRI suitability and to complete a simple reaction time task, during which their average reaction time was measured in order to appropriately set up the parameters for the experimental tasks to be completed in the scanner. This simple reaction time task was largely the same as the task to be completed in the scanner, with the exception that in this pre-test session, participants did not receive any feedback (positive, negative, or neutral, via body motion or text) in response to their reaction times. After this reaction time task, to ensure participants completely understood the tasks and the associations between the different cues and the types of feedback they would receive during the scanning task, participants received extensive training during this screening session, and were tested on their understanding afterwards. Participants also completed a demographic and health questionnaire, the Oldfield Handedness Inventory (Oldfield, 1971) (*M* = 73.71, *SD* = 19.35, corresponding to a right-handed sample), and the Autism Spectrum Quotient questionnaire (Baron-Cohen et al., 2001) (*M* = 14.76, *SD* = 6.85) corresponding to a mean value within the typical range for typically-developing individuals; Ruzich et al., 2015) during this screening session. Autistic traits were measured as a side project for a master's dissertation.

#### 2.4.2 Main experiment

During the scanning session for the main experiment, which occurred no later than one day after the screening session, participants were reminded of the task's different cue-outcome associations prior to entering the scanner. After performing all necessary safety checks, participants entered the scanner and completed two functional runs, a structural scan, and the remaining two functional runs. The researchers operating the scanner and speaking to the participants in between runs were careful not to praise participants throughout the duration of the two-part experiment, in order to avoid social satiation effects (Kohls et al., 2013; Gewirtz and Baer, 1958).

#### 2.4.3 Social incentive delay task

The Social Incentive Delay (SID) task is an adaptation of Knutson's (2000) Monetary Incentive Delay (MID) task. These tasks aim to investigate participants’ motivation to obtain rewards and avoid punishment. The version of the SID task used in the present study was split into two separate tasks: ‘Seeking Approval’ and ‘Avoiding Disapproval’ (Kohls et al., 2013). The SID is a simple reaction time task that examines both anticipatory and consummatory neural responses to appetitive and aversive stimuli.

**Fig. 2:**
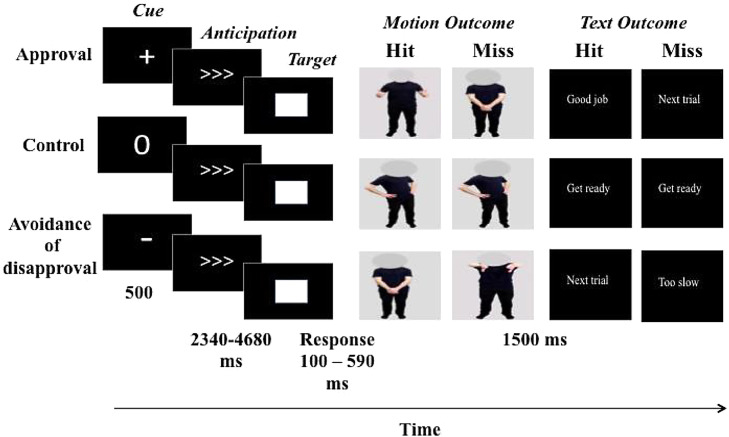
Illustration of the social incentive delay task, including the Seeking Approval and Avoiding Disapproval tasks. Approval and Avoidance trials were never presented within the same run. Participants were informed at the beginning of a block what type of feedback they would be receiving (motion or text), and intuitive cues were displayed at the beginning of a trial to show whether the trial was positive, negative, or a control.

Both the ‘Seeking Approval’ and ‘Avoiding Disapproval’ tasks consisted of 80 trials in total; 40 of these trials were incentive trials and 40 were control trials. Within each task, 40 of the trials provided feedback about performance in the form of human body motion and 40 provided feedback in the form of text. The experiment included a total of four runs, each approximately 13 min in length (2 Seeking Approval runs and 2 Avoiding Disapproval runs), following an event-related fMRI design. Before each run, participants were informed of the type of task they would be completing (i.e. Seeking Approval or Avoiding Disapproval), and at the beginning of each block of 20 trials within a run a screen was presented which informed participants whether they would be receiving feedback in the form of text or motion. Approval and disapproval trials were never presented within the same run; participants only ever saw approval and control, or disapproval and control trials within any given run. Within a run, trial type (incentive or control) was designated using intuitive cues (e.g. 0 for control trials, + for approval trials, and – for disapproval trials; Fig. 2).

During each trial, participants first saw a cue (500 ms), waited a variable interval (2340–4680 ms), and then responded to a white target square (100–590 ms) with a button press. Participants then received feedback (1500 ms) in the form of body gestures or text. In the Seeking Approval task, during the approval trials, target hits (responding to the white square within a predetermined timeframe) resulted in participants receiving feedback via either positive gestures, or positive text, and target misses (failing to respond to the white square within a predetermined timeframe) resulted in participants receiving feedback via neutral motion, or neutral text. During the control trials, both hits and misses resulted in participants receiving either neutral motion or neutral text feedback. In the Avoiding Disapproval task, during the disapproval trials, target hits resulted in participants receiving feedback via neutral motion or text, and target misses resulted in participants receiving feedback via negative motion or text (Fig. 2).

Following previous MID task designs, task difficulty was individually calibrated to the participants’ average reaction time (RT) (which was measured during the screening session), so that participants succeeded on approximately 60% of the trials. Further, in order to maintain the consistency of task difficulty, an online tracking algorithm was used to monitor and adjust the target duration in order to ensure that participants succeeded on approximately 60% of the trials (Kohls et al., 2013). Participants’ RT exceeded threshold 61% of the time in Run 1 and 2, 60% of the time in Run 3, and 63% of the time in Run 4.

At the end of the scanning session, participants completed two online surveys, similar to the surveys the independent group of participants completed in the pilot study to select the video and text stimuli. In these surveys, participants rated the emotional content of the video and text stimuli they encountered during the SID task, using a sliding scale from 0 to 100. These surveys were intended to ensure that the positive, negative, and neutral stimulus categories were rated significantly differently from each other, and that the motion and text stimuli were not rated differently.

### 2.5 Behavioral data analysis

We analyzed the behavioral RT data from the SID task completed in the scanner using a 2 (Task: Seeking Approval or Avoiding Disapproval) x 2 (Stimulus Type: Body Gestures or Text) x 2 (Trial: Incentive or Control) repeated measures ANOVA. Reaction times faster than 80 ms were removed from the data analyses, as these responses were unlikely to be under voluntary control; this resulted in 0.08% of responses being removed from further analyses.

Further, we analyzed the post-scan stimulus ratings using repeated-measures ANOVA, with post-hoc paired t-tests, and we also investigated whether differences emerged in the frequency of hits between the different conditions using a repeated-measures ANOVA.

### 2.6 fMRI

#### 2.6.1 Image acquisition

Images were collected using a 3 Tesla Philips Achieva MRI scanner with a SENSE phased-array 32-channel head coil, based in the Bangor Imaging Unit at Bangor University, Wales. Participants were asked to keep their heads as still as possible throughout the scanning session. Participants’ responses to the tasks were made via a scanner-compatible fibre-optic button box that enabled response times to be recorded. Participants viewed the trials on an MR safe screen positioned behind the scanner that was viewable to the participants via a mirror attached to the head-coil.

Functional data consisted of four 13-minute whole-brain T2* weighted echo-planar (EPI) sequences with 330 vol acquired per run (40 oblique axial slices, isotropic voxel size = 3.5 mm, TR/TE = 2340/30 ms, flip angle = 90°). A T1 weighted sequence collected in the same plane as the fMRI data was collected for the registration of the fMRI data to MNI space (number of slices = 40, slice thickness = 3.00 mm, TR/TE = 18/3.5 ms, flip angle = 8°). Two dummy scans were collected at the beginning of each run and were discarded from analyses due to the non-equilibrium state of magnetization.

### 2.7 fMRI data analysis

Image processing and statistical analyses were carried out using Statistical Parametric Mapping (SPM12: Wellcome Trust Centre for Neuroimaging, London; Friston, 2007) implemented with Matlab R2015a (MathWorks). For pre-processing, functional volumes for all participants were realigned, unwarped, slice-time corrected, and spatially smoothed using a Gaussian Kernel of 5 mm. Functional data were registered to MNI space.

The first-level analysis was conducted in SPM12. The first-level model for the within-run analyses of each task included regressors following a two (incentive: [Approval vs. Control] or [Avoidance vs. Control]), by two (phase: Anticipation or Outcome) by two (performance: Hit vs. Miss) by two (feedback: Motion vs. Text) design, resulting in 16 design matrix columns.

In accordance with Kohls et al. (2013), we modelled hit and miss trials separately, as VS/NAcc has been shown to respond more robustly when responses to reward-predicting cues are accurate (i.e. hits) compared to when these responses are inaccurate (i.e. misses) in rats (Francois et al., 2012). The anticipation phase was defined as the time between the onset of the trial cue and before the onset of the feedback. Regressors were convolved with a standard hemodynamic response function.

Whole-brain results were evaluated at *p* < 0.001, *k* > 20 voxels, uncorrected (Liebermann and Cunningham, 2009). Clusters were labelled using the IBASPM116 atlas (Lancaster et al., 1997, 2000; Maldjian et al., 2004, 2003; Tzourio-Mazoyer et al., 2002) (PickAtlas software, Wake Forest University, North Carolina, USA).

The fMRI objectives were as follows:

#### 2.7.1 Determine whether incentive trials activated reward regions more robustly than control trials during the anticipation of reward or avoidance of disapproval, for both body motion and text conditions independently

To address these questions, we ran four contrasts of (Approval > Control) *_motion_*, (Approval > Control) *_text_*, (Avoidance > Control) *_motion_*, and (Avoidance > Control) *_text_*. Additionally, to test our *a priori* hypothesis of greater NAcc involvement during social incentive motion and text trials compared to control trials, and during the motion compared to text condition, we performed region of interest (ROI) analyses within bilateral NAcc. The NAcc was anatomically defined using the WFU Pickatlas toolbox (http://www.fmri.wfubmc.edu/cms/software; Maldijian et al., 2003), and parameter estimates were extracted following a threshold-free cluster enhancement (pTFCE) (Spisák et al., 2019) method that improves detectability of neuroimaging signal by integrating cluster information into voxel-wise statistical inference. The *p*-values reported for these analyses are corrected for multiple comparisons across the two ROIs (left and right NAcc).

#### 2.7.2 Determine whether anticipating feedback presented via body gestures activates reward regions more strongly than the text condition

To achieve this, we evaluated the interaction between socially relevant feedback and feedback modality, separately for both approval and avoidance of disapproval tasks. This was calculated as (Approval *_motion_* > Control *_motion_*) > (Approval *_text_* > Control *_text_*) and (Avoidance *_motion_* > Control *_motion_*) > (Avoidance *_text_* > Control *_text_*), respectively.

#### 2.7.3 Investigate which regions are active during the anticipation of approval vs. the anticipation of avoidance of disapproval

These analyses were calculated as (Approval *_motion_* > Control *_motion_*) > (Avoidance *_motion_* > Control *_motion_*) and (Approval *_text_* > Control *_text_*) > (Avoidance *_text_* > Control *_text_*).

## 3 Results

### 3.1 Behavioral results

#### 3.1.1 Reaction time

**Fig. 3:**
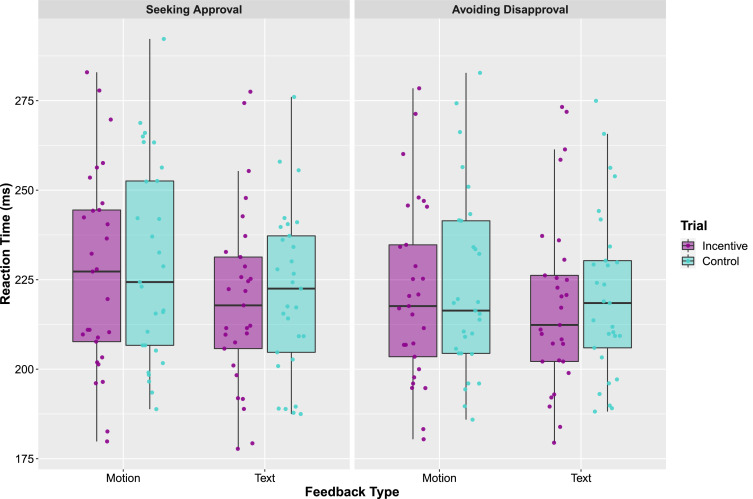
Reaction times for hits (in ms) for incentive and control trials, plotted separately for the two tasks (Seeking Approval and Avoiding Disapproval), and two feedback types (body motion and text). The points represent individual participants, the boxes represent the 25th and 75th percentiles, and the whiskers represent upper and lower values within 1.5*inter-quartile range.

The results from the repeated measures ANOVA (Fig. 3) revealed a main effect of trial type, showing that participants were faster to respond to the target during incentive trials (*M* = 221.43, SE = 4.53) compared to control trials (*M* = 223.81, SE = 4.40) (*F*(1, 28) = 7.90, *p* = 0.009, η^2^ = 0.22). This finding suggests that our incentive manipulations were successful. Additionally, a main effect of feedback type revealed that participants were faster to respond to the target during text trials (*M* = 219.96, SE = 4.26) compared to body motion trials (*M* = 225.28, SE = 4.70) (*F*(1, 28) = 20.97, *p* < 0.001, η^2^ = 0.43). A significant interaction between task and feedback type (*F*(1, 28) = 6.87, *p* = 0.014, η^2^ = 0.20) demonstrated that participants responded faster to the target during the text feedback compared to the body gesture feedback in the Seeking Approval task, while they responded equally quickly during motion and text trials in the Avoiding Disapproval task.

#### 3.1.2 Frequency of hits

**Fig. 4:**
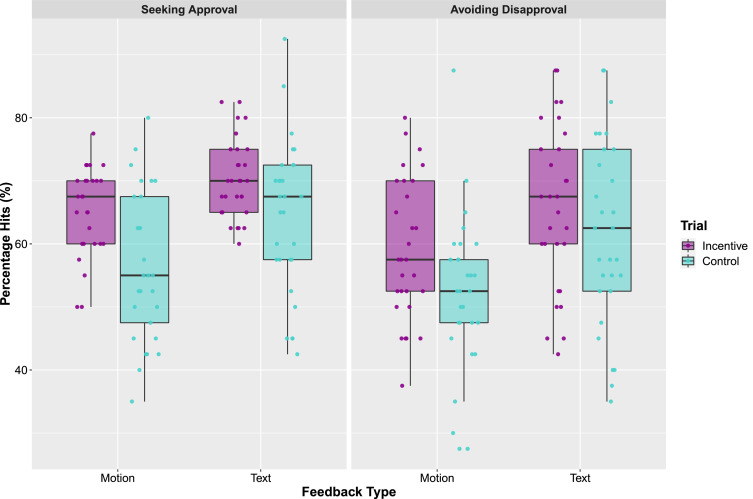
Illustrates the percentage of hit trials across the different conditions. The points represent individual participants, the boxes represent the 25th and 75th percentiles, and the whiskers represent upper and lower values within 1.5*inter-quartile range.

To investigate whether any significant differences emerged in the number of hits (i.e. correct responses) between the different conditions, a 2 × 2 × 2 ANOVA was performed (Fig. 4). This analysis revealed a main effect of Task (*F*(1, 28 = 5.38, *p* = 0.028, η^2^ = 0.16) such that participants obtained more hits during the Seeking Approval task (*M* = 65.00, *SE* = 1.14) than in the Avoiding Disapproval task (*M* = 59.63, *SE* = 1.98). There was also a main effect of Trial type (*F*(1, 28) = 19.61, *p* < 0.001, η^2^ = 0.41), with more hits observed during incentive trials (*M* = 65.19, *SE* = 1.23) than control trials (*M* = 58.53, *SE* = 1.73). Further, there was a main effect of Feedback Type (*F*(1, 28) = 33.81, *p* < 0.001, η^2^ = 0.55) demonstrating that participants were more successful during the text (*M* = 65.56, *SE* = 1.68) compared to the body motion condition (*M* = 58.17, *SE* = 1.18), which supports the reaction time findings presented above.

#### 3.1.3 Post-scan stimulus ratings

**Fig. 5:**
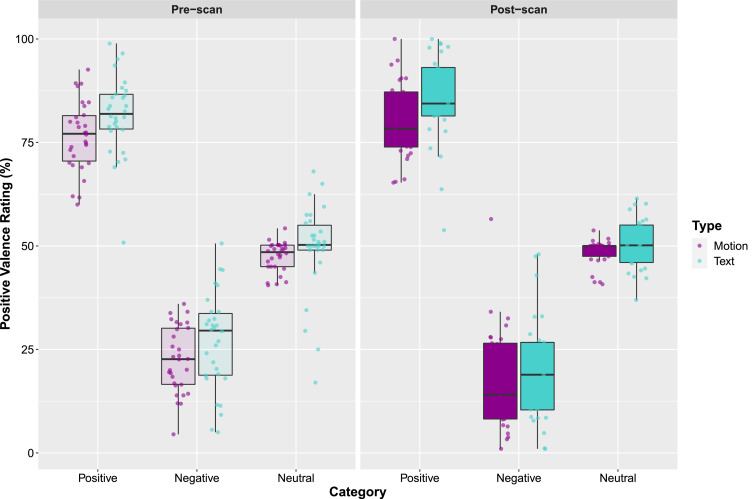
Illustrates the ratings of the stimuli pre- and post-scan. The pre-scan ratings were provided by an independent group of participants who did not participate in the fMRI study. The points represent individual participants, the boxes represent the 25th and 75th percentiles, and the whiskers represent upper and lower values within 1.5*inter-quartile range.

A repeated measures ANOVA investigating the post-scan stimulus ratings (Fig. 5) revealed that the three stimulus categories (positive, negative, and neutral) for both the video stimuli (*F*(1.17, 32.76) = 277.51, *p* < 0.001, η^2^ = 0.91) and the text stimuli (*F*(1.20, 33.72) = 243.96, *p* < 0.001, η^2^ = 0.90 were rated significantly differently from each other (all Sidak-corrected comparisons had *p*-values of < 0.001). However, although an independent group of participants prior to the scanning study did not rate the motion and text stimuli as being different from each other, paired t-tests (corrected for multiple comparisons) revealed that the participant sample who underwent fMRI rated positive text stimuli (*M* = 85.37, *SE* = 2.01) significantly more positive than the positive video stimuli (*M* = 80.40, *SE* = 1.70; *t* (28) = −2.77, *p* = 0.010), and the neutral text stimuli (*M* = 50.97, *SE* = 0.78) more positive than the neutral motion stimuli (*M* = 48.53, *SE* = 0.60), post-scan.

### 3.2 fMRI results

#### 3.2.1 Incentive vs. control trials

##### 3.2.1.1 Seeking approval

To investigate brain regions activated more robustly during incentive trials compared to control trials during the anticipation of approval, we ran simple effect contrasts comparing approval trials separately for the motion and text conditions: (Approval > Control) *_motion_*, and (Approval > Control) *_text_*.

**Fig. 6:**
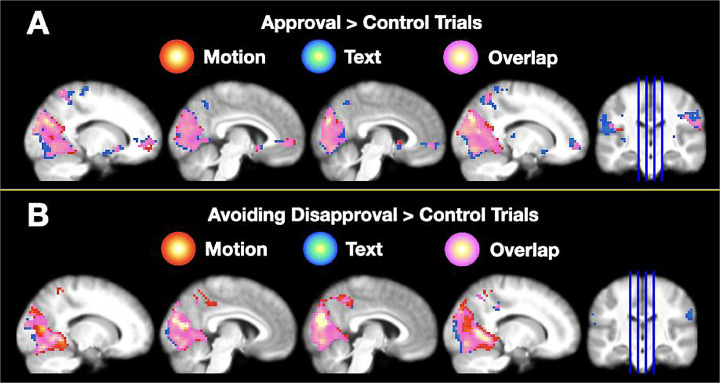
A. Group analysis for the Approval > Control contrast for both motion and text feedback. B. Group analysis for the Avoiding Disapproval > Control contrast for both motion and text feedback. All clusters presented are thresholded at *p* < 0.05 FDR_corrected_ and shown on a group-averaged T1-weighted image in MNI space.

*Motion:* The whole-brain analysis revealed several clusters of activation (Table 1 & Fig. 6A) including the cuneus, left superior temporal gyrus, left ACC and right caudate. The ROI analysis (Table 2 & Fig. 8) confirmed that the left and right NAcc were more strongly activated during the anticipation of approval than during the anticipation of control feedback provided via body motion, replicating earlier studies that showed participants images and videos of faces as rewarding feedback (Kohls et al., 2013; Rademacher et al., 2010; Spreckelmeyer et al., 2009).

**Table 1:** Results from the whole brain analysis for the different condition contrasts. This table lists the brain regions that emerge at a threshold of *p* < 0.001, *k* = 20 uncorrected. Regions indicated with bold font signify clusters significant at the *p* < 0.05 FDR_corrected_ threshold.

	Region	BA	MNI Coordinates			*t*-value	Cluster Size	*P*_FDR-Corrected_
			x	y	z			
	*APPR > CON _motion_*							
**R**	**Cuneus**	**19**	**12**	**−79**	**31**	**9.63**	**4008**	**< 0.001**
**L**	**Superior temporal gyrus**	**22**	**−57**	**−31**	**16**	**6.52**	**88**	**0.004**
**L**	**Superior parietal lobule**	**7**	**−18**	**−55**	**61**	**6.33**	**123**	**0.001**
**L**	**ACC**	**32**	**−9**	**44**	**−5**	**6.03**	**163**	**< 0.001**
**L**	**Postcentral gyrus**	**6**	**−51**	**−10**	**31**	**5.68**	**123**	**0.001**
**R**	**Superior parietal lobule**	**7**	**21**	**−52**	**64**	**5.66**	**175**	**< 0.001**
R	Superior fronto-orbital gyrus	45	24	20	−14	4.89	24	0.183
**R**	**Caudate**	**48**	**6**	**11**	**1**	**4.54**	**50**	**0.031**
	*APPR > CON _text_*							
**L**	**Superior occipital gyrus**	**19**	**−18**	**−73**	**22**	**9.37**	**3347**	**< 0.001**
**R**	**Postcentral gyrus**	**1**	**60**	**−13**	**25**	**6.82**	**920**	**< 0.001**
**L**	**Supramarginal gyrus**	**40**	**−57**	**−25**	**19**	**6.09**	**422**	**< 0.001**
**L**	**ACC**	**10**	**−12**	**44**	**−5**	**6.60**	**232**	**< 0.001**
**R**	**Superior parietal lobule**	**7**	**18**	**−61**	**52**	**5.99**	**276**	**< 0.001**
**L**	**Superior parietal lobule**	**7**	**−21**	**−55**	**61**	**5.67**	**191**	**< 0.001**
**R**	**Precentral gyrus**	**4**	**24**	**−13**	**52**	**5.31**	**44**	**0.035**
**L**	**Putamen**	**8**	**−21**	**17**	**−11**	**4.93**	**80**	**0.004**
L	Precentral gyrus	4	−18	−13	61	4.92	28	0.107
**R**	**Superior orbito-frontal gyrus**	**45**	**24**	**17**	**−14**	**4.81**	**90**	**0.003**
	*AVOI > CON _motion_*							
**R**	**Mid temporal gyrus**	**37**	**48**	**−61**	**4**	**11.61**	**4210**	**< 0.001**
**R**	**Precuneus**	**31**	**6**	**−49**	**46**	**5.89**	**221**	**< 0.001**
R	Hippocampus		36	−13	−11	4.79	34	0.218
	*AVOI > CON _text_*							
**L**	**Cuneus**	**18**	**0**	**−85**	**16**	**7.85**	**2446**	**< 0.001**
**R**	**Mid occipital gyrus**	**19**	**42**	**−76**	**16**	**5.85**	**162**	**< 0.001**
**L**	**Mid occipital gyrus**	**19**	**−39**	**−82**	**16**	**5.54**	**233**	**< 0.001**
**R**	**Supramarginal gyrus**	**40**	**60**	**−22**	**25**	**5.38**	**85**	**0.005**
R	Precentral gyrus	24	60	8	19	5.13	24	0.191
**L**	**Supramarginal gyrus**	**31**	**−60**	**−28**	**37**	**4.98**	**78**	**0.006**
R	Insula	40	36	−13	40	4.82	24	0.191
**R**	**Mid Cingulum**	**40**	**15**	**−34**	**40**	**4.39**	**97**	**0.003**
R	Insula	40	42	−13	−5	4.07	24	0.191
	*APPR > CON _motion_* vs*. APPR > CON _text_*							
**R**	**IFG**	**19**	**45**	**−70**	**−5**	**8.09**	**234**	**< 0.001**
**L**	**Mid occipital gyrus**	**19**	**−48**	**−79**	**4**	**6.49**	**73**	**0.008**
	*APPR > CON _text_* vs*. APPR > CON _motion_*							
**L**	**Superior occipital gyrus**		**−15**	**−91**	**1**	**6.31**	**90**	**0.010**
L	Paracentral lobule	6	−15	−16	64	5.50	49	0.070
R	Lingual gyrus	18	15	−82	−14	5.33	26	0.174
R	Cuneus	19	18	−91	34	4.97	34	0.147
L	Precentral gyrus	6	−54	−1	34	4.24	28	0.174
L	Superior frontal gyrus	6	−21	2	46	4.16	23	0.191
	*AVOI > CON _motion_* vs*. AVOI > CON _text_*							
**R**	**Mid temporal gyrus**	**19**	**51**	**−70**	**−2**	**9.66**	**354**	**< 0.001**
**L**	**Mid occipital gyrus**	**19**	**−51**	**−76**	**1**	**6.50**	**104**	**0.001**
	*AVOI > CON _text_* vs*. AVOI > CON _motion_*							
**R**	**Calcarine**	**18**	**18**	**−91**	**1**	**7.35**	**56**	**0.029**
**L**	**Mid occipital gyrus**	**18**	**−15**	**−91**	**−8**	**5.76**	**48**	**0.029**

Bold font indicates *p*-values less than 0.05 FDR_corrected_.

**Table 2:** Results from the ROI analysis for the different contrasts.

*Left NAcc*			
	*Estimate*	*t*-value	*P _Corrected_*
**APPR > CON *_motion_***	**0.48**	**3.55**	**0.002**
**APPR > CON *_text_***	**0.60**	**4.37**	**<0.001**
AVOI > CON *_motion_*	0.13	0.81	0.856
AVOI > CON *_text_*	0.21	1.39	0.352

*Right NAcc*			
	*Estimate*	*t*-value	*P _Corrected_*
**APPR > CON *_motion_***	**0.35**	**2.63**	**0.028**
**APPR > CON *_text_***	**0.53**	**3.89**	**0.002**
AVOI > CON *_motion_*	0.20	1.96	0.120
**AVOI > CON *_text_***	**0.34**	**2.63**	**0.028**

Bold font indicates *p*-values less than 0.05.

*Text:* Similar regions to those activated during the Approval > Control *_motion_* were activated during the Approval > Control *_text_* contrast (Table 1 & Fig. 6A), including a cluster comprising the left superior occipital gyrus, left ACC, and a cluster comprising the left putamen. The ROI analysis (Table 2 & Fig. 8) confirmed that the bilateral NAcc was more strongly activated during incentive trials compared to control trials.

##### 3.2.1.2 Avoiding disapproval

To examine participants’ neural responses during incentive trials compared to control trials during the anticipated avoidance of social disapproval, we first ran simple effect contrasts of Avoidance > Control *_motion_*, and Avoidance > Control *_text_*.

*Motion:* The whole-brain analysis for the Avoidance > Control *_motion_* contrast (Table 1 & Fig. 6B) revealed a large cluster of activation centered on the right mid temporal gyrus and precuneus. The ROI analysis did not identify greater engagement of the NAcc during the anticipated avoidance of disapproval than during the anticipation of control feedback via body motion (Fig. 8).

*Text:* The Avoidance > Control *_text_* contrast revealed several significant clusters of activation, including a cluster centered on the cuneus. The ROI analysis revealed a significant increase in activation in the right NAcc, but not left NAcc, during incentive trials compared to control trials (Fig. 8).

#### 3.2.2 Comparison between body motion and text feedback

##### 3.2.2.3 Seeking approval

**Fig. 7:**
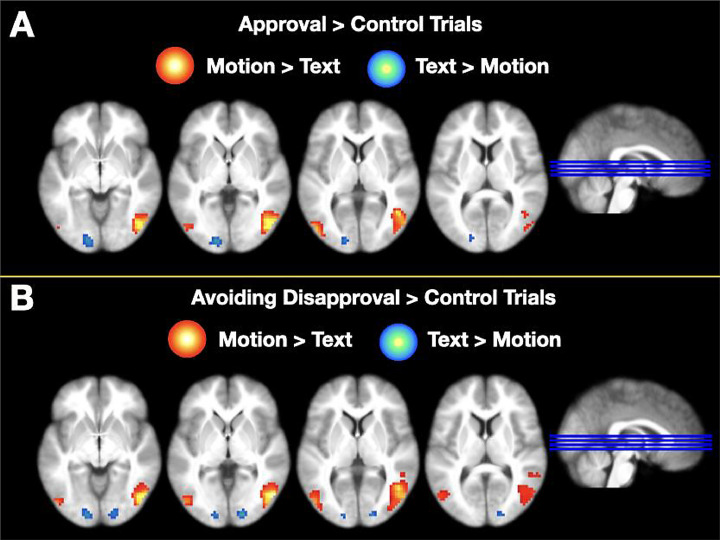
Group analysis for the motion > text, and text > motion contrast for the Seeking Approval task (A) and the Avoiding Disapproval task (B). All clusters presented are thresholded at *p* < 0.05 FDR_corrected_ and shown on a group-averaged T1-weighted image in MNI space.

To investigate whether any brain regions responded more robustly to body motion than text feedback in the approval compared to control conditions in the Seeking Approval task we next calculated (Approval *_motion_* > Control *_motion_*) > (Approval *_text_* > Control *_text_*) (Table 1 & Fig. 7A). This analysis revealed that the left inferior frontal gyrus and mid occipital gyrus were more strongly activated during the body motion compared to text condition. The inverse contrast revealed that the superior occipital gyrus was more sensitive to the anticipation of text compared to body motion feedback. These findings demonstrate that differences in visual processing emerge between the body motion and text conditions during the anticipation period, even when the visual stimuli are the same for both conditions during this time.

**Fig. 8:**
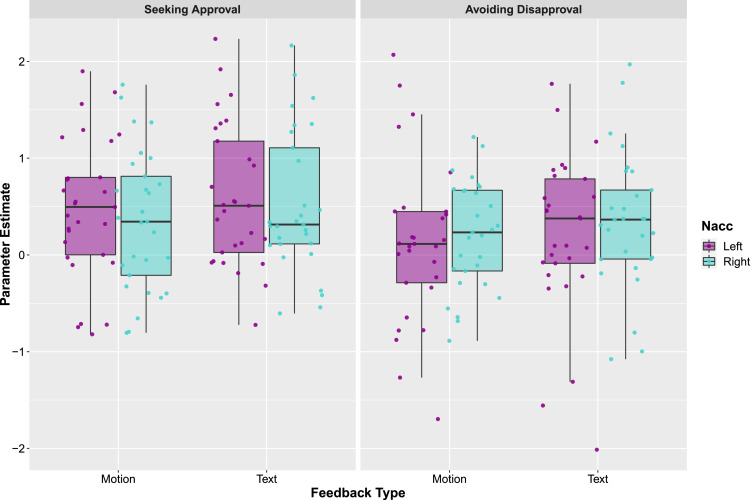
Results from the ROI analysis investigating bilateral NAcc activity during the incentive > control contrasts. The points represent individual participants, the boxes represent the 25th and 75th percentiles, and the whiskers represent upper and lower values within 1.5*inter-quartile range. Similar to a previous study by Kohls et al. (2013), the magnitude of NAcc activation differed substantially across participants.

##### 3.2.2.4 Avoiding disapproval

We also investigated whether any activation differences were found between the body motion and text trials during the Avoiding Disapproval task. This was calculated as (Avoidance *_motion_* > Control *_motion_*) > (Avoidance *_text_* > Control *_text_*) (Table 1 & Fig. 7B). The Motion > Text analysis revealed that the mid temporal and mid occipital gyri were more strongly activated during the motion condition than the text condition. The inverse contrast revealed significant activations in the calcarine sulcus and the mid occipital gyrus. Similarly to the seeking approval contrasts, these findings demonstrate the differences in visual processing between anticipating body motion and text.

#### 3.2.3 Comparison between anticipating approval and avoidance of disapproval

We also investigated whether any activation differences emerged between the two tasks, calculated separately for motion and text conditions. These analyses were calculated as (Approval *_motion_* > Control *_motion_*) > (Avoidance *_motion_* > Control *_motion_*) and (Approval *_text_* > Control *_text_*) > (Avoidance *_text_* > Control *_text_*) (and their inverse contrasts). No significant activation differences emerged when contrasting Seeking Approval and Avoiding Disapproval, similarly to the findings of Kohls et al. (2013).

## 4 Discussion

The overarching aim of the present study was to investigate the extent to which the anticipation of rewarding feedback presented via body gestures activates brain regions associated with reward processing in a similar manner to what previous studies have demonstrated with social feedback presented via faces. Further, guided by the Social Motivation theory (Chevallier et al., 2012), we hypothesized that social feedback presented via body gestures would activate brain regions associated with reward processing more robustly than a less social control condition presenting feedback via text.

We found that participants were faster to respond to targets in the text feedback compared to the motion feedback condition in the social approval task. While this finding was unexpected, the valence ratings for the stimuli, obtained from participants following the fMRI portion of the study, suggest that this particular participant sample perceived the positive text stimuli as more positive than the positive motion stimuli. Although the motion and text stimuli were piloted prior to the imaging study by an independent group of participants to ensure they were not perceived as more or less positive than each other, due to natural variation in participant samples, it was not possible to ensure that the participants who took part in the imaging study would perceive the stimuli in an identical way. Additionally, we found that participants obtained more hits (i.e. correct responses) during the text condition than the body motion condition, which might also be explained by the differences in stimulus ratings given by participants post-scan. The increased saliency of the positive text stimuli could lead to increased motivation in participants to obtain these rewards, which could explain why participants showed faster reaction times, and more hits, during the text condition. However, although what participants saw on screen during the anticipation phase is the same for both motion and text trials, it could still be the case that participants are processing the anticipation of text feedback faster than the anticipation of motion feedback, which resulted in the different behavioral responses to the target cue that we report here.

### 4.1 Seeking approval task

The neuroimaging findings demonstrated that anticipation of both body motion and text feedback recruited similar brain regions, including the postcentral and superior parietal gyri, and the ACC (which has been implicated in reward anticipation and action-outcome associations; Knutson et al., 2001, Shima and Tanji, 1998, Hayden and Platt, 2010). Other areas implicated in reward anticipation, namely the cuneus (Nestor et al., 2010; Spreckelmeyer et al., 2009) and caudate, were recruited during the anticipation of rewarding body gestures. Brain areas involved in reward anticipation, including the putamen, were also engaged during the anticipation of rewarding feedback via text (Breiter et al., 2001; Kohls et al., 2013). The ROI analysis also demonstrated that the bilateral NAcc was more strongly activated during the anticipation of both incentive body gestures and incentive text feedback compared to neutral feedback, in the approval task. These findings are consistent with, and complement, previous research that shows these regions are active during anticipation of social feedback presented via faces (Kohls et al., 2013) and non-social feedback, such as money (Spreckelmeyer et al., 2009).

Although the behavioral findings suggest that participants were more motivated to obtain positive feedback presented via text than motion feedback, the fMRI findings demonstrate that both kinds of feedback recruited reward regions similarly. Our findings thus demonstrate that social information, such as feedback presented via body motion alone, can act as a rewarding stimulus.

We hypothesized that feedback presented via body gestures would be perceived as more rewarding than text feedback, as we know from prior work that we can read a wealth of social information from bodies alone (de Gelder, 2006; Greven et al., 2019), even without visual access to the face, and that participants value this type of social stimulus and are willing to exert effort to engage with it (Williams and Cross, 2018). However, we did not find the expected increased activation in reward-related regions during the anticipation of feedback presented via motion compared to text. Anticipating body gestures or text feedback activated reward regions similarly, and no differences were found in NAcc activation magnitude between the two feedback modalities. Previous research has shown that NAcc activation is mediated by the saliency of a reward (Knutson et al., 2001; Spreckelmeyer et al., 2009), thus our findings suggest that feedback presented via body motion and text were perceived as equally salient, given the similar pattern of neural engagement. However, our post-scan stimulus ratings demonstrate that participants perceived the text stimuli as more positive than the motion stimuli. A possible explanation for this is that feedback provided via text is perceived as less ambiguous than feedback provided via body gestures; in other words, it is possible that the meaning behind gestures is more difficult to understand. Nonetheless, our study demonstrates that social feedback presented via body motion alone is a powerful social stimulus that motivates behavior and engages reward-related brain regions, similarly to feedback presented via faces (Kohls et al., 2013; Spreckelmeyer et al., 2009; Rademacher et al., 2010), money (Dichter et al., 2012), and drugs (Childress et al., 1999).

### 4.2 Avoiding disapproval task

We also included an Avoiding Disapproval task (Kohls et al., 2013) to investigate whether avoiding punishment results in similar patterns of brain activity as anticipating rewards. We hypothesized that anticipating the avoidance of disapproval presented via body gestures or text would activate areas involved in reward anticipation. However, only the middle temporal gyrus and right precuneus survived cluster correction in the Incentive > Control contrast for the body gesture condition. Areas such as the left cuneus, mid occipital gyrus, and mid cingulum were engaged during the anticipation of avoidance of disapproval via text. In the ROI analysis, we found no increased activation of the left NAcc to incentive compared to control trials, and the right NAcc was more strongly activated only for the text condition. Kohls et al. (2013) found engagement of the NAcc for both approval and disapproval tasks. It is unclear why we did not find the hypothesized activation of the left NAcc for the disapproval task in our sample of participants. However, it is possible that participants were not responding very differently to the incentive and control trials in the motion condition as this condition showed the smallest difference in RT (incentive RT – control RT). Although it is plausible that brain regions implicated in reward processing, such as the NAcc, would be engaged during these conditions due to the opportunity to avoid punishment being inherently rewarding (Skinner, 1938; Shimojo, & O'Doherty, 2006), we found engagement of the right NAcc only in the text condition. Findings regarding NAcc involvement in the anticipation of avoidance of punishment are mixed, with some studies demonstrating increased activation to anticipating avoidance of monetary loss compared to a control condition (Carter et al., 2009; Delgado et al., 2009), and some reporting no differences between conditions (Knutson et al., 2001; 2003). To our knowledge, only one other study to date has investigated NAcc activity in response to social punishment (Kohls et al., 2013), thus underscoring the value of further research on the neurocognitive correlates of social punishment.

The magnitude of NAcc activation across the different feedback modalities (motion and text) and tasks (seeking approval and avoiding disapproval) varied considerably between participants, which is in accordance with previous literature involving monetary (Carter et al., 2009) and social (Kohls et al., 2013) incentives. These findings thus suggest large individual differences in the motivational value of social approval and disapproval, although it is possible these differences could be attributed to noise (such as small variations or imperfections in co-location of the NAcc across participants in MNI space). It would be beneficial for future studies, with appropriately powered sample sizes, to systematically investigate individual differences in reward sensitivity. Further, disorders such as Autism Spectrum Condition (ASC) are characterized by a reduction in social motivation (Chevallier et al., 2012), and previous studies have demonstrated decreased activation of the VS/NAcc in response to social rewards in individuals with ASC (Scott-Van Zeeland et al., 2010) and those reporting more autistic traits (Hsu et al., 2018). Thus, differences in reward responsivity in the SID task can arise from differences in reward sensitivity across participants. A challenge for future studies is to investigate these questions in clinical disorders such as ASC to determine whether these individuals also demonstrate reduced motivation and reduced activation of the VS/NAcc when engaging with social stimuli other than faces. Previous work has already identified dysfunction in biological motion processing systems in ASC (Kaiser et al., 2010), and that neural signatures in brain circuits involved in biological motion processing and social motivation/reward predict intervention effectiveness in children with ASC (Yang et al., 2016). Although we measured autistic traits in our sample of 31 participants, we do not believe we had an adequate sample size and enough power to run correlational analyses on reward sensitivity and autistic traits (however, our data are available for any researcher wishing to examine questions related to autistic traits).

The design of our tasks provided useful feedback for incentive trials (e.g. positive feedback for target hits, and neutral feedback for misses) in both the social approval and the avoidance of social disapproval tasks; however, control feedback trials were uninformative (i.e. neutral feedback for both hits and misses). Thus, the inclusion of feedback about task performance in the incentive trials but not the control trials could have contributed to the behavioral and neural differences between incentive and control trials. Future research could include feedback about task performance in the incentive and the control trials, in order to ensure the emerging differences are not only due to whether participants receive feedback or not.

### 4.3 Limitations and future directions

While the use of dynamic videos in this study can be viewed as a step toward greater ecological validity or naturalism when studying the neurocognitive architecture supporting social perception, we nonetheless acknowledge that considerable room for improvement remains regarding our particular stimuli. Various parameters, such as the emotional valence and the motion energy of the videos, were controlled for, however, this may have resulted in a reduction in how natural the gestures presented in the videos appeared. Thus, future studies should aim to strike a better balance between good stimulus control and ecological validity (perhaps achieved through the use of virtual reality, as a number of researchers are now exploring; Pan and Hamilton, 2018, Tarr et al., 2018). Another broader point that research in this domain should consider going forward is how personal social perception is, and how individual differences shape brain responses when watching (and interacting) with other people. As Dubois and Adolphs (2016) convincingly argue, our understanding of the relationship between brain responses, psychological traits and behavior will be significantly advanced through a greater focus on these individual differences. With future studies moving in the direction of considering and parsing the heterogeneity in brain responses, this should move cognitive neuroscience research towards a research model similar to precision medicine, with the many benefits this approach entails (c.f., Chung et al., 2020; Seligson et al., 2020). This kind of approach would also shed light on the large variability seen in NAcc activation in the current study.

In conclusion, our results complement and extend, previous research investigating the neural engagement of reward-related regions in response to social feedback. The results revealed that the NAcc was engaged during the anticipation of rewarding feedback presented via body motion alone, demonstrating that this type of stimulus can motivate behavior in a similar way to feedback presented via faces (Kohls et al., 2013; Spreckelmeyer et al., 2009; Rademacher et al., 2010) and money (Dichter et al., 2012). In the real world, our daily lives involve socially engaging with other people moving around us, and we can read a wealth of social information from other people's body motion and gestures even when we cannot clearly see their facial features. The present study findings advance our understanding of the types of feedback we find rewarding and the neural underpinnings of social motivation in the typical population.

## CRediT authorship contribution statement

**Elin H. Williams:** Conceptualization, Methodology, Investigation, Formal analysis, Data curation, Writing - original draft. **Laura Bilbao-Broch:** Investigation. **Paul E. Downing:** Conceptualization, Writing - review & editing. **Emily S. Cross:** Conceptualization, Methodology, Visualization, Writing - review & editing, Supervision, Funding acquisition.

## Appendix Supplementary materials

Image, application 1

Image, application 2

## Footnotes

1 For completeness and to inform future investigations, we also include analyses from the “consumption” (or “outcome”) phase in our Supplementary Materials.
