# Supplementary material for: Examining the value of body gestures in social reward contexts
Source: Neuroimage. 2020 Nov 15;222:117276. doi: 10.1016/j.neuroimage.2020.117276 (PMC7779365; doi:10.1016/j.neuroimage.2020.117276)
Supplement: Supplementary file 2 [file mmc2.docx]

***Supplemental Analysis: Outcome phase analysis***

Although the central questions addressed in this study concerned the anticipation of reward, we present results from the outcome phase model here for a more complete view of the data (Supplementary Table 1). The outcome phase analysis aimed to achieve 3 primary objectives:

1. The first question concerned whether incentive feedback activated reward regions more robustly than control feedback during both approval and avoidance tasks, for both body motion and text conditions. To address this, we ran contrasts for (Approval > Control) *_motion_*, (Avoidance > Control) *_motion_*, (Approval > Control) *_text_*, and (Avoidance > Control) *_text_* for the outcome phase (from video onset to offset).
2. Next, we were interested in investigating whether viewing feedback presented via motion would activate reward regions more strongly than the text condition. Therefore, our next analyses evaluated whether differences in activation emerged when viewing feedback presented via motion compared to text (Motion > Text) for both task types (approval vs avoidance) separately.
3. We were also interested in investigating which regions are active during appraisal of approval and successful avoidance of disapproval, for both motion and text conditions separately. These analyses were calculated as (Approval *_motion_* > Control *_motion_*) > (Avoidance *_motion_* > Control *_motion_*) and (Approval *_text_* > Control *_text_*) > (Avoidance *_text_* > Control *_text_*).
4. Incentive vs. Control trials

*Seeking Social Approval*

The Approval > Control *_motion_* contrast for the outcome phase did not reveal any clusters of activation surviving threshold (*p* < 0.001, *k* = 20). However, the same contrast for the text condition revealed a cluster in the right ACC.

*Avoiding Social Disapproval*

The Avoidance > Control *_motion_* contrast did not reveal any clusters of activation surviving threshold. The Avoidance > Control *_text_* contrast revealed a cluster in the right precuneus, however this did not survive FDR-correction.

1. Comparison between body motion and text feedback

*Seeking Approval:* To investigate whether any brain regions responded more robustly to body motion than text feedback in the approval compared to control conditions in the Seeking Approval task we next calculated (Approval *_motion_* > Control *_motion_*) > (Approval *_text_* > Control *_text_*). No clusters emerged at the p < 0.001, k = 20 threshold for the motion > text contrast, but the inverse contrast revealed a cluster surviving FDR-correction in the mid temporal gyrus (Supplementary Table 2).

*Avoiding Disapproval:* To investigate whether any brain regions responded more robustly to body motion than text feedback in the disapproval compared to control conditions in the Avoiding Disapproval task we next calculated (Avoidance *_motion_* > Control *_motion_*) > (Avoidance *_text_* > Control *_text_*). No clusters survived threshold for the motion > text contrast, but the inverse contrast revealed clusters in the superior temporal and mid temporal gyri.

1. Comparison between anticipating approval and avoidance of disapproval

*Comparison between seeking approval and avoiding disapproval tasks:* We investigated whether any differences in activation emerged when participants received positive feedback in the Approval task compared to avoiding negative feedback in the Avoidance task. For the motion condition, the Approval > Avoidance analysis revealed several clusters of activation, including a cluster in the hippocampus. However, no clusters survived threshold for the inverse contrast. No significant clusters emerged for the Approval > Avoidance contrast for the text condition.

*Supplementary Table 1. Results from the whole brain analysis for the outcome phase contrasts. This table lists the brain regions that emerge at a threshold of p < 0.001, k = 20.*

|  | Region | BA | MNI Coordinates | | | *t*-value | Cluster Size | *P*_FDR-Corrected_ |
| --- | --- | --- | --- | --- | --- | --- | --- | --- |
|  |  |  | x | y | z |  |  |  |
|  |  |  |  |  |  |  |  |  |
|  | *Approval > Control Text* |  |  |  |  |  |  |  |
|  |  |  |  |  |  |  |  |  |
| **R** | **ACC** | ***32*** | **6** | **41** | **-2** | **4.81** | **55** | **0.035** |
|  |  |  |  |  |  |  |  |  |
|  | *Avoidance > Control Text* |  |  |  |  |  |  |  |
|  |  |  |  |  |  |  |  |  |
| R | Precuneus | *7* | 6 | -64 | 61 | 4.62 | 23 | 0.704 |
|  |  |  |  |  |  |  |  |  |
|  | *(Approval >Control Text) > (Approval > Control Motion)* | | | |  |  |  |  |
|  |  |  |  |  |  |  |  |  |
| **R** | **Mid temporal gyrus** | 19 | **42** | **-70** | **7** | **5.15** | **186** | **0.001** |
| L | Superior occipital gyrus | 18 | -9 | -97 | 4 | 4.79 | 26 | 0.334 |
| L | Calcarine | 18 | -3 | -85 | -14 | 4.44 | 24 | 0.334 |
|  |  |  |  |  |  |  |  |  |
|  | *(Avoidance >Control Text) > (Avoidance > Control Motion)* | | | |  |  |  |  |
|  |  |  |  |  |  |  |  |  |
| **R** | **Superior temporal gyrus** | **22** | **60** | **-37** | **13** | **6.34** | **110** | **0.01** |
| **R** | **Mid temporal gyrus** | **19** | **42** | **-67** | **7** | **5.69** | **201** | **0.001** |
| L | Superior occipital gyrus | 18 | -9 | -97 | 1 | 4.67 | 38 | 0.136 |
| L | Mid temporal gyrus |  | -36 | -67 | 10 | 4.47 | 63 | 0.051 |
| L | Calcarine | 18 | -3 | -85 | -14 | 4.42 | 31 | 0.162 |
|  |  |  |  |  |  |  |  |  |
|  | *(Approval >Control Motion) > (Avoidance > Control Motion)* | | | |  |  |  |  |
|  |  |  |  |  |  |  |  |  |
| **L** | **Hippocampus** | **54** | **-18** | **-10** | **-23** | **7.13** | **38** | **0.043** |
| **L** | **Lingual gyrus** |  | **-9** | **-34** | **-8** | **6.72** | **40** | **0.043** |
| **L** | **Fusiform gyrus** | **37** | **-39** | **-55** | **-14** | **5.48** | **89** | **0.004** |
| **R** | **Inferior temporal gyrus** | **37** | **45** | **-43** | **-20** | **5.45** | **100** | **0.003** |
| **R** | **Mid temporal gyrus** |  | **45** | **5** | **-26** | **5.39** | **126** | **0.002** |
| **R** | **Lingual gyrus** | **36** | **9** | **-34** | **-8** | **5.06** | **58** | **0.016** |
| **L** | **Superior temporal pole** |  | **-39** | **11** | **-20** | **4.9** | **80** | **0.006** |
| **L** | **Mid cingulum** | **23** | **-12** | **-40** | **31** | **4.35** | **39** | **0.043** |
| **L** | **ACC** |  | **-3** | **20** | **16** | **4.24** | **42** | **0.043** |
| **L** | **Mid temporal gyrus** | **39** | **-45** | **-58** | **10** | **4.08** | **59** | **0.016** |
|  |  |  |  |  |  |  |  |  |

Bold font indicates *p*-values less than 0.05 FDR_corrected_
